# The Role of a Ketogenic Diet in the Treatment of Dementia in Type 2 Diabetes Mellitus

**DOI:** 10.3390/nu15081971

**Published:** 2023-04-19

**Authors:** Lin Bai, Yue Zhou, Jie Zhang, Junpeng Ma

**Affiliations:** 1 Department of Neurosurgery, West China Hospital of Sichuan University, Chengdu 610041, China; 2 Key Laboratory of Transplant Engineering and Immunology, NHFPC, West China Hospital of Sichuan University, Chengdu 610041, China; 3 Core Facility of West China Hospital of Sichuan University, Chengdu 610041, China; 4 Department of Pharmacy, Xindu District People’s Hospital of Chengdu, Chengdu 610500, China

**Keywords:** type 2 diabetes mellitus, dementia, ketogenic diet, neuroprotective

## Abstract

Type 2 diabetes mellitus (T2DM) shares a common molecular mechanism and underlying pathology with dementia, and studies indicate that dementia is widespread in people with T2DM. Currently, T2DM-induced cognitive impairment is characterized by altered insulin and cerebral glucose metabolism, leading to a shorter life span. Increasing evidence indicates that nutritional and metabolic treatments can possibly alleviate these issues, as there is a lack of efficient preventative and treatment methods. The ketogenic diet (KD) is a very high-fat, low-carbohydrate diet that induces ketosis in the body by producing a fasting-like effect, and neurons in the aged brain are protected from damage by ketone bodies. Moreover, the creation of ketone bodies may improve brain neuronal function, decrease inflammatory expression and reactive oxygen species (ROS) production, and restore neuronal metabolism. As a result, the KD has drawn attention as a potential treatment for neurological diseases, such as T2DM-induced dementia. This review aims to examine the role of the KD in the prevention of dementia risk in T2DM patients and to outline specific aspects of the neuroprotective effects of the KD, providing a rationale for the implementation of dietary interventions as a therapeutic strategy for T2DM-induced dementia in the future.

## 1. Introduction

Diabetes mellitus (DM) is a metabolic disorder caused by decreased insulin production and is characterized by persistent hyperglycemia and abnormalities in carbohydrate, lipid, and protein metabolism. By 2045, it is projected that 783 million individuals between the ages of 20 and 79 will have diabetes; this number is increased from the approximately 537 million individuals with diabetes in 2021 [1]. There are two distinct forms of DM, and each has a unique frequency and pathogenesis. Type 1 DM (T1DM) accounts for 5–10% of all occurrences of diabetes and is characterized by the death of pancreatic cells by T-cell-mediated immunity. Type 2 DM (T2DM) is characterized by insulin resistance and accounts for 90% of all cases of diabetes [2,3]. Because different parts of the brain are vulnerable to changes in the balance of insulin and glucose, both T1DM and T2DM lead to impairments in the central nervous system (CNS) to varying degrees. However, T2DM is associated with a 50% higher risk of cognitive impairment than T1DM [4].

T2DM is currently the most prevalent type of metabolic illness caused by atypical insulin regulation. T2DM and other metabolic disorders that impact glucose homeostasis may increase the risk of developing certain neurodegenerative illnesses [5,6]. In addition, T2DM has been linked to oxidative stress, inflammation, synapse loss, and decreased neural plasticity in the CNS, which may lead to a neurodegenerative process that is characterized by cognitive impairment and memory loss [7,8]. According to epidemiological research, people with T2DM have a higher incidence of cognitive impairment, including Alzheimer’s disease (AD), than people without the disease [5]. Finally, the population trend for dementia is quite similar to that seen in T2DM, and as a result, DM and dementia co-occur more frequently than would be expected by chance alone [9].

## 2. Diabetes Mellitus and Cognitive Disability

### 2.1. Dementia and Cognitive Impairment

According to an epidemiological study, more than 55 million individuals worldwide have dementia, and more than 10 million new cases are diagnosed each year [10]. Memory, reasoning, orientation, comprehension, computation, learning capacity, language, and judgement are just a few of the processes that dementia impacts. For instance, the frontal and temporal lobes of the brain are more frequently affected by Tau protein accumulation and plaque development, which may cause dementia and result in irreversible neuronal cell death [11]. Moreover, Parkinson’s disease results in an aberrant build-up of alpha-synuclein in the neurons of the substantia nigra, which causes Lewy body dementia [12]. Blood clots, malformed blood arteries, and abnormal brain tissue are the causes of vascular dementia [13].

Dementia associated with several brain regions has surpassed heart disease as the primary cause of death in people with DM [14]. Individuals with DM, in contrast to the general population, have an increased risk of developing dementia, particularly vascular dementia. Many systematic studies that compared people with DM to those without DM reported summary relative risks (SRRs) of 2.38 (95% confidence interval (CI) 1.79–3.18) for vascular dementia and 1.39 (95% CI 1.16–1.66) for AD [15]. Nineteen population-based studies that included 44,714 persons, 6184 of whom had DM, revealed similar outcomes and an RR of 1.21 (95% CI 1.02–1.45) for mild cognitive impairment [16]. Those with DM had higher odds of developing dementia from any cause compared to people without the condition, according to two meta-analyses of prospective cohort studies [17,18]. Furthermore, it was demonstrated that T2DM accelerates the development of dementia in patients with mild cognitive impairment [19]. Another significant finding revealed that individuals with T2DM had a 45% prevalence of mild cognitive impairment, compared to a stated prevalence of 3–22% for the general population [20]. However, it should be noted that the frequency of moderate cognitive impairment in those with T2DM was similar in those under 60 (46%) and those over 60 (44%); these findings are in contrast to those of other studies that suggested that mild cognitive impairment was more prevalent in older persons, particularly those over 65 [21]. However, a different meta-analysis showed that there was cognitive decline among T2DM patients under the age of 65, indicating that there may be a burden of cognitive disease among younger individuals with DM [22].

### 2.2. T2DM and Dementia

**Figure 1:**
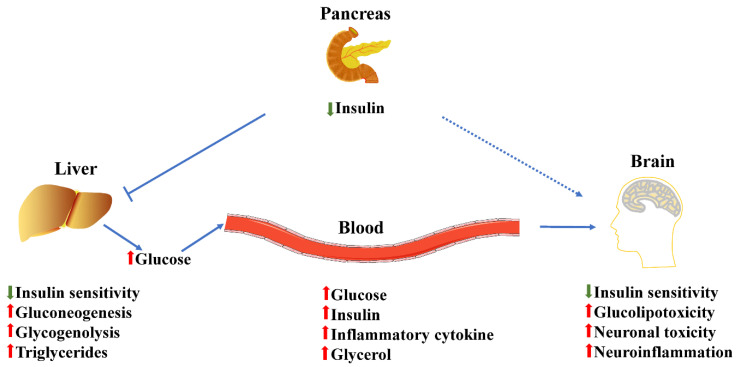
Impaired blood glucose metabolism in T2DM-induced dementia. Patients with dementia from T2DM exhibit systemic hyperglycemia, hyperlipidemia, and hyperinsulinemia. Decreases in insulin resistance and insulin sensitivity are characteristics of T2DM. A lack of insulin sensitivity prevents the liver from absorbing and using glucose from the blood. Defective glucose absorption encourages the liver to speed up gluconeogenesis and glycogenolysis, which raises the level of glucose in the blood. Decreased glucose uptake also causes a dependency on fatty acid metabolism as the main source of energy production. Hepatic fatty acids also encourage the synthesis of triglycerides and ketone bodies, and reactive oxygen species and oxidative imbalance are caused by excess glucose in hepatic mitochondria. In addition, the passive removal of materials from the blood is inhibited by capillarization and inflammatory cytokine release, both of which are promoted by oxidative stress. Finally, the brain also displays insulin resistance with excess glucose through blood, which contributes to increasing neuronal damage and neuroinflammation.

T2DM and dementia are age-related conditions that impact millions of people worldwide. Blood glucose levels in T2DM individuals with dementia are abnormal, and they are 1.5–2.5 times more likely to experience neurological problems than people without diabetes. Compared to patients without diabetes, patients with diabetes have a higher prevalence of AD, which is also linked to a higher incidence or fatality rate [12]. DM is associated with insulin resistance (IR), and dysregulation in the molecular mechanism of insulin production may result in histopathological abnormalities in DM. A significant risk factor for dementia and cognitive decline is hyperglycemia, which may also have a deleterious effect on cognitive performance. Individuals with T2DM are prone to develop dementia and other neurological conditions, but sporadic dementia tends to be more prevalent [23]. The brain uses a glucose homeostasis mechanism to manage the energy level throughout the body. It has been found that up to 80% of dementia patients exhibit glucose intolerance [24]. During more than 11 years of research, scientists have discovered a higher prevalence of dementia and AD, as well as a 50–100% higher chance of acquiring dementia, in people with diabetes [25]. Memory loss, difficulty concentrating, difficulty with routine tasks, altered behavior, and confusion about time and location are all common dementia symptoms [9]. The relationship of T2DM and dementia is shown in Figure 1.

## 3. Overview of a Ketogenic Diet

**Figure 2:**
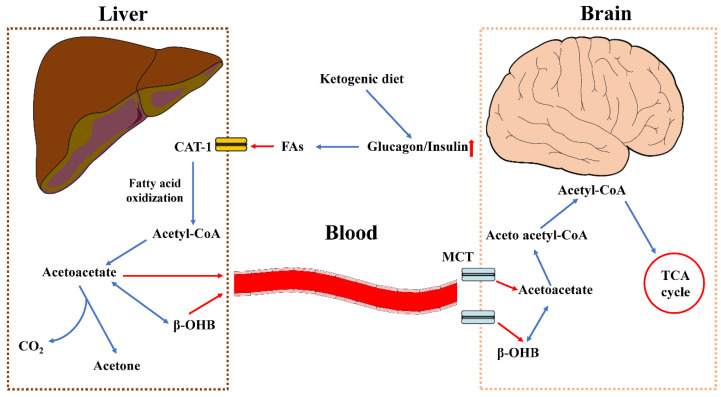
Illustration of the biochemistry of ketogenesis in the liver and brain. Long-term glucose restriction causes the ratio of glucagon to insulin to rise, which causes the release of free fatty acids into the blood. Carnitine acylcarnitine translocase-1 (CAT-1) transports free fatty acids into liver mitochondria, where they are utilized to oxidize fatty acids to produce acetyl coenzyme A (acetyl-CoA). The production of ketone bodies allows these molecules to start the ketogenesis process. Acetyl-CoA is transformed into acetoacetate, which then permits the reversible reduction to acetone and β-hydroxybutyrate (β-OHB). These ketone bodies subsequently leave the liver and travel through the circulation to reach peripheral tissues and the brain, where they are carried in the brain by monocarboxylic acid transporters. In the brain, β-OHB can be changed back into acetoacetate, acting as a potential source of acetyl-CoA to release energy through the tricarboxylic acid cycle. Abbreviations: Acetyl-CoA, acetyl coenzyme A; β-OHB, β-hydroxybutyrate; CAT, carnitine acylcarnitine translocase; CO_2_, carbon dioxide; FAs, fatty acids; MCT, monocarboxylic acid transporter; TCA, tricarboxylic acid.

A high-fat, low-carb diet with the right quantities of protein, vitamins, and minerals is known as a ketogenic diet (KD). Under normal physiological conditions, the body prefers to consume carbs, which are broken down into glucose and distributed throughout the body to provide energy, but this diet stimulates the body to consume fats more readily. It has been determined that the KD may be implemented to treat neurodegenerative and neuropsychiatric diseases [26,27]. This high-fat, moderate-protein, low-carb diet releases ketone bodies (principally β–hydroxybutyrate (β-OHB) and acetate) from the breakdown of fat that act as an alternative fuel, shifting away from the use of glucose as the body’s primary energy source. The KD was first employed by doctors as a treatment for epilepsy in the 1920s [26]. By following a sustained KD, as opposed to starvation, one can reach a degree of nutritional ketosis that is much below that of pathological ketoacidosis [28]. For example, the resting β-OHB concentration was elevated to approximately 2.0 Mm after aerobic exercise, and during a 1 h recovery period, β-OHB decreased to 0.85 mM after the nutritional ketogenic diet. These findings suggest that short-term nutritional ketosis does not impair aerobic exercise capacity, which may be due to increased utilization of β-OHB when carbohydrate stores are diminished [29]. The brain has evolved to use ketones to protect and enhance crucial central functions in situations of glucose restriction or elevated energy needs [30]. This is particularly true during periods of fasting during sleep, when circulating ketone bodies, particularly β-OHB, can be increased and maintained by ketones. The symptoms of some age-related disorders have been found to improve with higher levels of β-OHB [31], thus offering justification for the creation of therapeutic ketogenic treatments for neurodegenerative disorders [32]. The biochemistry of ketogenesis in the liver and brain is shown in the Figure 2.

The KD and other dietary therapies have been studied as novel therapeutic modalities, primarily for the treatment of T2DM. In numerous studies, the KD has also been demonstrated to decrease insulin resistance and enhance glucose tolerance in an animal model [33] and in patients with T2DM [34]. Recent studies have, nevertheless, emphasized the crucial functions of the KD in the treatment of a number of neurological illnesses [35,36,37]. Researchers have recently focused on the neuroprotective properties of the KD. Despite mounting evidence that food therapy is effective, the precise mechanism underlying its protective effects is yet unknown. In this review, we evaluated the experimental and clinical evidence and examined the processes underlying the neuroprotective benefits of the KD, proposing that the application of a KD might be a feasible therapeutic option for T2DM-induced dementia based on its neuroprotective qualities.

## 4. Neuroprotective Effects of the Ketogenic Diet

### 4.1. Modulating Brain Neurotransmitters

When there is a lack of nutrients after exercise or when there are not enough carbohydrates available, brain neurons use ketone bodies as a source of energy. Gluconeogenesis, the tricarboxylic acid cycle, and fatty acid β-oxidation are connected to the regulation of ketone bodies [38]. Gamma-aminobutyric acid (GABA) and glutamate neurotransmitter activity have been shown to decrease under hyperglycemic circumstances, and cholinergic transmission has been discovered to be dysregulated in the hippocampus [39]. In patients with T2DM, the levels of dopamine and its receptors are decreased [40]. Serotonin and dopamine are associated with sadness and anxiety and are regulated by KB, which also increases the levels of GABA and excitatory glutamate [41]. Moreover, ketone bodies participate in cell survival and neural anti-apoptotic mechanisms [41]. The underlying mechanisms are related to how ketone bodies control brain signaling, boost insulin sensitivity, decrease the consequences of oxidative stress, boost synaptic activity, and keep neurotransmitter activity at a constant level. Indeed, we are aware that low ketone body levels could result in pathogenic T2DM situations [42]. However, the modulation of ketone bodies at various levels in neuroprotection and neurotoxicity in diabetes-induced dementia therapeutic strategies requires more study.

### 4.2. Modulating β-Amyloid

Malfunctions in the respiratory chain and mitochondria might affect the metabolism of amyloid precursor protein (APP), producing hazardous β-peptide (Aβ) [43]. The KD offers ketones as substitute metabolic substrates for the brain, perhaps alleviating the effects of impaired glucose metabolism [44]. Moreover, KD may aid in reducing the development of amyloid plaques by reversing the toxicity of Aβ (1–42) [44,45]. Exogenous β-OHB-treated mice exhibited better mitochondrial performance, decreased brain Aβ levels, and protection from amyloid toxicity [46]. After just 40 days of treatment, the KD led to decreased soluble Aβ deposit levels in transgenic mice with AD by 25% [47]. However, we should note that the existence of the ApoE4 genotype in humans, which is a risk factor for the development of AD, may influence this process [48].

### 4.3. Modulating the Blood–Brain Barrier

In both T1DM and T2DM mouse models, hyperglycemia, blood–brain barrier (BBB) permeability, and cognitive impairment are strongly associated with each other [49]. Because the BBB maintains brain homeostasis and limits access of hazardous substances and infections to the brain, its disruption can impair cognitive function by causing abnormal molecular transport between the peripheral circulation and the brain [50]. Although there is currently no conclusive evidence, given the current state of our knowledge, it is reasonable to approach this subject from a prospective viewpoint. At the BBB, membrane disruption can also occur, similar to what occurs in the intestinal barrier. For instance, this disruption occurs in epilepsy, AD, and other neurological illnesses [51,52]. The BBB becomes more permeable to β-OHB when blood ketones are present in higher concentrations [53]. Ketosis can lead to the restoration of BBB integrity, which increases the amount of connexin-43 (Cx43), monocarboxylate transporters (MCTs), and glucose transporters (GLUT transporters) that are used to create the barrier [54,55]. Furthermore, a KD increases the concentration of proteins involved in clearing out amyloid plaques, including glycoprotein P (P-gp) and phosphatidylinositol-binding clathrin assembly protein (PICALM), which facilitates the outflow of the aforementioned amyloid plaques across the BBB [56].

### 4.4. Maintaining the Brain Energy Supply

When there is relatively little energy available, a KD can boost metabolic efficiency and keep the overall metabolic quantity steady, improving the ability of neurons to resist damage. Studies have shown that ketones are a more effective energy source than glucose. They are digested more quickly than glucose and can enter the tricarboxylic acid cycle without passing through the glycolytic pathway [57,58]. Glycolysis and fatty acid production are suppressed as a result of the peroxisome proliferator-activated receptor (PPAR) being activated by fatty acids [59]. A KD increases ATP generation through mitochondrial oxidation while decreasing glycolytic ATP production [59], which enhances oxidative mitochondrial metabolism, resulting in downstream metabolic changes. The key factors leading to protection against neuronal death by apoptosis and necrosis include ketosis, higher serum fat levels, and lower serum glucose levels. For instance, earlier research found that a KD influences the overexpression of genes in the hippocampus region that encode enzymes involved in mitochondrial and energy metabolism [60]. Hence, by offering alternative energy substrates, therapeutic ketosis might be seen as a type of metabolic therapy. These metabolic alterations enhance brain metabolism and restore mitochondrial ATP synthesis. Moreover, after KD treatment, reduced formation of reactive oxygen species (ROS), antioxidant benefits, reduced inflammatory response, and increased activity of neurotrophic factors are seen [58]. The stability of synaptic activity across neurons is also a result of elevated amounts of Krebs cycle intermediates, elevated GABA to glutamate ratios, and activated ATP-sensitive potassium channels [58].

### 4.5. Restoring Cardiometabolic Function

Previous research has provided evidence for a possible connection between cerebrovascular function and cognitive function [61]. Only one study has examined the relationship between cerebrovascular function and cognition in adults, and the results showed a link between cerebrovascular function and overall cognition in older women [62]. Over the course of two years, a study compared the effects of the KD to standard therapy in patients with T2DM and discovered significant gains in restoring cardiometabolic function while using less medication [63]. This was demonstrated by decreases in lipids, blood pressure, body mass index (BMI), fasting glucose, and fasting insulin in the KD group. Diabetes was also resolved in the KD group but not in the control group (53.5% reversal, 17.6% remission). Several studies examining the effects after 10 weeks and one year revealed similar decreases in BMI and medication use when comparing a KD to conventional therapy in T2DM patients [64,65]. Furthermore, comparable encouraging cardiometabolic alterations were discovered in a recent five-year clinical trial of the KD in T2DM patients, indicating the possibility of positive long-term effects. Finally, it has been discovered that a KD reverses the rise in tiny low-density lipoprotein (LDL) particles, a frequent feature of diabetic dyslipidemia [66]. Hence, it has been suggested that these beneficial cardiometabolic alterations have lowered the risk of cardiovascular disease in the T2DM population, while further study is required to offer solid proof.

### 4.6. Modulating Oxidative Stress

Mitochondria are also a major source and target of reactive oxygen species (ROS). The initial form of ROS is superoxide, which is later converted to hydrogen peroxide (H_2_O_2_). Mitochondrial dysfunction produces excessive ROS and reduces the mitochondrial electron transport chain (mETC) activity and ATP synthesis [67]. Moreover, mitochondrial DNA (mtDNA)-encoding respiratory chain complexes are susceptible to ROS, resulting in oxidative damage and mutations of mtDNA. This further damages the function of mETC and aggravates energy failure and oxidative stress [68]. In T2DM, enhanced mitochondrial ROS levels have also been observed to activate the apoptotic cascade by triggering the release of cytochrome c, leading to neuronal apoptosis and impaired cognition [69,70]. In T2DM, oxidative stress also induces a novel form of iron-mediated cell death via phospholipid peroxidation and ferroptosis. In hippocampal neurons of mice, transferrin receptor 1 levels are upregulated, the levels of ferroportin-1 and ferritin heavy chain are decreased, the expression of mitochondrial ferritin is decreased, and the expression of mitoferrin was increased, suggesting hippocampal neuronal and mitochondrial iron overload [71,72]. In addition, excess Fe^2+^ can react with H_2_O_2_ to generate hydroxyl radicals with stronger oxidative ability through the Fenton reaction and undergo a lipid peroxidation reaction with unsaturated fatty acids [73]. Elevated mitochondrial ROS and decreased glutathione peroxidase activity lead to the accumulation of lipid peroxides, which trigger ferroptosis and cognitive deficits in hippocampal neurons in T2DM [72].

A KD increases ketone synthesis in the liver and reduces blood glucose levels. The oxidation of fatty acids, especially polyunsaturated fatty acids (PUFAs), is the primary cause of the rise after a KD [74]. PUFAs control the membrane receptors in neurons, activate peroxidase by inhibiting voltage-gated sodium and calcium channels, and upregulate the production of mitochondrial uncoupling protein (UCP). The uncoupling procedure lowers the voltage of the mitochondrial membrane, which eventually lowers the generation of ROS [75,76]. β-OHB is said to scavenge ROS, but acetate does so when the concentration of ROS exceeds the physiological range (IC50 20–67 mM) [77]. A frequent mechanism linked to β-OHB is the positive effects on the redox potential of the electron transport chain [78]. Only β-OHB and acetate can stop ATP decline in neurons, even though all ketones (β-OHB and acetate) can minimize ROS accumulation and neuronal cell death caused by the suppression of glycolysis [79,80]. In addition, acetate did not have this impact on an in vivo model of hypoglycemia where β-OHB protected hippocampal lipid peroxidation [81,82]. The most notable modifications were seen in the hippocampus. The glutathione peroxidase levels and total antioxidant capabilities were increased, according to in vivo experiments in which mice were fed a KD. In a clinical study, a KD increased glutathione in the brains of epileptic children, suggesting a pivotal role of glutathione in the antioxidant neuroprotective effect of the KD in the human brain [83]. The mechanism by which a KD increases GSH may involve the upregulation of nuclear factor erythroid 2-related factor 2 (Nrf2) transcription factor, which is a primary responder to cellular stress that promotes glutathione biosynthesis in rats [84]. The upregulation of Nrf2 may depend on the mild oxidative and electrophilic stress initially induced by the KD, leading to chronic cellular adaptation, the induction of protective proteins, and stable improvements in the redox state [84]. However, another clinical study showed that ketone bodies may reduce reactive oxygen species without increasing GSH [8]. This difference might depend on GSH homeostasis impairment due to the particular experimental settings for the in vitro study [83]. In animal models of brain damage, a KD was able to activate the Nrf2 pathway, and Nrf2 was then transported into the nucleus. This increased the expression of the downstream antioxidant protein heme oxygenase-1 (HO-1), which is regarded as one of the most crucial components for preventing oxidative stress [85]. In a study using an ischaemic stroke model, ketone therapy following brief middle cerebral artery (MCA) blockade improved mitochondrial activity and decreased oxidative stress, thereby lowering the infarct amount and enhancing neurological function after ischaemic stroke. The increase in NAD+-dependent Sirtuin 3 (SIRT3) and its downstream substrates, superoxide dismutase 2 (SOD2) and forkhead box O3A (FOXO3A), in the penumbra area was responsible for neuroprotective effects [86]. However, a recent study using transgenic mice with defective mitochondrial DNA repair found histological evidence of neurological degeneration linked to the KD, despite a rise in mitochondrial biogenesis and antioxidant markers [87]. Additional contradictory evidence revealed that prolonged exposure to high ketone concentrations can cause oxidative damage. High dosages of β-OHB or acetate may cause lipid peroxidation, nitric oxide production, and reduced expression of SOD, glutathione peroxidase, and catalase, according to research performed on calf hepatocytes. Another study, for instance, found that acetate, but not β-OHB, stimulated the mitogen-activated protein kinase (MAPK) pathway in rat hepatocytes [88,89]. Thus, the role of the KD in oxidative stress needs more research.

### 4.7. Modulating Inflammatory Responses

Inflammation and oxidative stress are two crucial components of AD neuropathology that underlie neurotoxic processes that result in neuronal death in the parts of the brain involved in memory and cognitive functions [90,91], which is caused by the release of proinflammatory cytokines and NO and the suppression of neurotrophins [90]. A KD has varying and inconsistent ways through which it can regulate inflammatory responses and immune cell activities [55]. Long-term nutrient restriction may lessen inflammation [92]. T1DM-induced persistent ketosis is regarded as a proinflammatory state [93]. The fundamental reason why β-OHB has an impact on inflammation is that the monocytes and macrophages of the immune system express large amounts of GPR109A. High concentrations of ketones counteract the anti-inflammatory effects of β-OHB [94,95]. The G protein-coupled receptor GPR109A, also known as the hydroxy-carboxylic acid 2 (HCA2) receptor, is found on neutrophils, macrophages, and adipocytes. According to multiple prior studies, GPR109A ligands have anti-inflammatory effects on obesity, atherosclerosis, neurological disorders, inflammatory bowel diseases, and different types of cancer [96]. It was discovered that retinal pigment epithelium (RPE) cells from individuals and animals with diabetes have increased GPR109A expression [95]. Genetic deletion or pharmacological suppression of GPR109A negated the anti-inflammatory effects of β-OHB in RPE cells. Overexpression of GPR109A could augment such effects [94]. Earlier research proposed that the actions of β-OHB on HCA2 receptors could be the mechanism through which KD exerts its neuroprotective benefits [36]. These findings demonstrated that following a distal MCA blockage, animals fed a KD or given β-OHB through subcutaneous minipumps had reduced ischemic infarct volumes. This effect was not present in HCAR2-null mice. Moreover, β-OHB reduced proinflammatory proteins (COX-2, iNOS) or released cytokines (IL-1, IL-6, TNF, CCL2/MCP-1) in LPS- or TNF-induced inflammation, in part by reducing NF-κB translocation [97]. Nevertheless, the GPR109A-dependent protection afforded by β-OHB in neurodegenerative inflammatory responses did not entail inflammatory mediators, such MAPK pathway activation (ERK, JNK, or P38), but instead may necessitate COX-1-dependent PGD2 synthesis [36,95]. It is interesting to note that although the ability of macrophages to inhibit the NLRP3 inflammasome via β-OHB was discovered to be independent of GPR109A, this ability is essential in ischemic stroke rats [92].

The KD also exerts effects on inflammatory processes by inhibiting the activation of the NF-kB light-chain enhancer of activated B cells [36]. It causes an uptick in the immunological response and the downregulation of COX2 and inducible nitric oxide synthase [98], and cytokines, such IL-1b, IL-6, CCL2/MCP-1, and TNF-α, are less active [99]. Moreover, peroxisome proliferator-activated receptor (PPAR) can decrease the production of NF-kB, hence reducing the neuronal damage caused by N-methyl-D-excitotoxicity aspartate (NMDA) [100]. Additionally, the KD exerts anti-inflammatory actions by inhibiting the activation of microglial cells [101], which produce higher levels of neuroprotective mediators, such as neurotrophins (such as neurotrophin-3 (NT-3), brain-derived neurotrophic factor (BDNF), and glial cell line-derived neurotrophic factor (GDNF)), and increases the proapoptotic characteristics and levels of molecular chaperones (proteins preventing the aggregation of polypeptides into potentially toxic molecules) [102,103]. The enhanced expression of inflammatory cytokines can be reversed by β-OHB [76]. Finally, we speculate that one of the most significant diabetes-induced dementia-modifying effects of a KD may be lowering inflammation.

## 5. Summary

Abnormalities in cognitive function can develop as a side effect in both T1DM and T2DM patients. However, those with a history of T2DM experience this cognitive dysfunction more severely and frequently. The KD has been utilized for many years to treat a variety of neurological conditions, and a sizable number of studies have recently confirmed the function of the KD in neuroprotection. By lowering oxidative stress, regulating energy metabolism, controlling inflammation, and affecting other potential processes, it may exert neuroprotective effects. It is inevitable that all neurological diseases will impact human health through oxidative damage, energy metabolism issues, or inflammatory responses, even though the precise processes of the KD in the treatment of neurological diseases are still unknown. Many mechanisms are frequently involved in neurological illnesses, and the KD may also contribute to regulating these pathways. This review explores the complex role of a KD application in dementia caused by T2DM and its potential working mechanisms. There are many variables, including hyperglycemia, obesity, neuroinflammation, oxidative stress, and Aβ plaques, and each has a separate or combined impact on cognitive impairments. Here, we have covered in great length how the KD affects cognitive function in the brain, facilitating disease treatment and enhancing patient symptoms and quality of life. The KD has great application potential in clinical settings. To improve the method and effectiveness of KD therapy, which can better prevent or possibly reverse T2DM-associated dementia, future studies are required to define the roles of the components in KD, their therapeutic targets, and related pathways.

## Data Availability

All data are available upon request.
